# Comparison of the accuracy of guided implant surgery between two implant-planning software: a retrospective cohort study

**DOI:** 10.3389/froh.2025.1729521

**Published:** 2026-01-22

**Authors:** Wantong Zhou, Guiyan Feng, Zhilin Luo, Lianyi Xu, Yingguang Cao, Ke Song

**Affiliations:** 1 Department of Stomatology, Tongji Hospital, Tongji Medical College, Huazhong University of Science and Technology, Wuhan, China; 2 Department of Prosthodontics and Implantology, School of Stomatology, Tongji Medical College, Huazhong University of Science and Technology, Wuhan, China; 3 Hubei Province Key Laboratory of Oral and Maxillofacial Development and Regeneration, Wuhan, China; 4 School of Stomatology, Tongji Medical College, Huazhong University of Science and Technology, Wuhan, China

**Keywords:** 3Shape Implant Studio, computer-aided implant surgery, digital workflow, GuideMia, implant-planning software

## Abstract

**Purpose:**

To evaluate the difference of a single posterior implant of the same length between planned and actual positions for two commonly used static implant planning software packages following a tooth-supported partially guided surgery protocol.

**Materials and methods:**

There are 75 implant placement cases were included in this retrospective study. 40 were designed using the GuideMia Implant System, and 35 were designed using 3Shape Implant Studio. The implant position on the postoperative cone-beam computed tomography was superimposed on the planned implant position. Coronal, apical, and angular deviations in the 3D direction were measured for each group using an additional evaluation software program. Six risk factors that may influence the accuracy were evaluated separately: jaw, location, implant system, missing teeth at the free end, and implant length and diameter. Linear regression models were established to analyze the source of deviation.

**Results:**

No statistically significant differences were observed between the two implant planning software programs no matter the implant length is 8 mm, 10 mm, or 12 mm (*P* > 0.05). Significant differences were observed at the entry point (*P* = 0.003), apex (*P* = 0.005), and angle (*P* = 0.002) between the free and non-free ends.

**Conclusions:**

The implant planning programs showed similar results regarding the implant position accuracy of the same length. However, an implant located at the free end of a single missing posterior tooth has a significant influence on the accuracy.

## 1 Introduction

Computer-guided implant surgery involves integrating cross-disciplinary research that includes computer-aided design and manufacturing (CAD/CAM) technology, radiographic imaging technology, and three-dimensional (3D) printing technology aimed to achieve “prosthetic-driven” precision implantation (1, 2). mplant planning software is a digital tool that provides clinicians with the registration of digital images and 3D structural information that can be manipulated to complete the corresponding preoperative design (3, 4). These data will be used for surgical guide printing to assist clinicians in accurately and safely executing implant placement to avoid damage to the adjacent tooth roots, mandibular nerves, and maxillary sinuses. Therefore, software with higher accuracy can provide more reliable and precise measurements, allowing for the optimal placement of implants in relation to adjacent anatomical structures and the desired prosthetic outcome (5). Especially for cases with multiple missing or edentulous teeth, significant implant angular deviation leads to considerable difficulties to the subsequent restoration and may cause biomechanical complications (6). Various clinical studies have demonstrated that computer-aided implant surgery reduces patient discomfort and results in more accurate implant positioning than that obtained via conventional freehand surgery (7–9). Two types of guided implant surgery systems exist—static (template-based) and dynamic navigation. Presently, static systems are more popular because of their lower cost and space requirements compared to dynamic navigation (10–12). Static systems can be further divided into partially guided systems (guided drilling) and fully guided systems (guided drilling and guided implant placement). Partially guided templates allow clinicians to evaluate and adjust the final drilling, inserted angulation, and implant depth, permitting corrections of deviations during the procedure. However, partially guided may exhibit greater variation in implant deviation compared to fully guided, particularly with respect to distal zones and angular placement (4, 13).

In 1998, the Colombia Technology Company launched the first commercial computer-aided dental implant planning software, SimPlant, which enabled preoperative planning and design for guided implant surgery (14). Since then, multiple software companies have developed their implant planning software, such as SimPlantTM (Materialize, Leuven, Belgium), Nobel GuideTM (Nobel Biocare, Gothenburg, Sweden), coDiagnostiX (Dental Wings GmbH), GuideMia Implant Studio (GuideMia Technologies), and Implant Studio (3Shape) (3, 15). However, the accuracy of implant planning software can be affected by various factors, such as the matching accuracy of cone-beam computed tomography (CBCT), intraoral optical scanning (IOS) data, and 3D reconstruction and software algorithms (16, 17). Therefore, a comprehensive comparison of different digital software programs is necessary to identify the strengths and weaknesses of each system and determine which system is most suitable for specific clinical scenarios (18–20). Despite numerous studies investigating the accuracy of different software, research to compare their clinical effects and accuracy in single-tooth implant placements is limited (21, 22). And when considering the placement of dental implants, the length of the implant is a critical factor that must be carefully assessed. While longer implants may seem like a good idea for providing greater stability and support, research has indicated that there is an increased potential for placement deviations with longer implants. Therefore, this retrospective cohort study aimed to evaluate the accuracy between the planned and placed positions for two-implant planning software programs that are most commonly used in clinical practice, explore the implantation discrepancy caused by different implant planning software, provide references for rational selection in clinical practice, and examine risk factors influencing the accuracy of the digital workflow of implant insertion. The null hypothesis was that no significant difference would be found in the clinical accuracy of the planned and placed implant positions between the two-implant planning software programs.

## 2 Materials and methods

Prior to this retrospective cohort study, a survey was conducted on the frequency of different implant planning software programs in seven dental laboratories in Wuhan, China, between 2020 and 2023. The results showed that of the 13,200 cases designed using different software, the 3Shape Implant Studio was used in 7,165 cases, accounting for 54%, and the GuideMia implant system was used in 4,705 cases, accounting for 35%. This suggested that the 3Shape Implant Studio and GuideMia implant system are the most commonly used implant planting software (Table 1).

**Table 1:** The frequency of different implant planning software programs between 2019 and 2023.

Category	Software	Usage proportion (%)
All implant planning software that was used in dental laboratories	3Shape implant studio	54
	GuideMia implant system	35
	coDiagnostiX	5
	SimPlant^TM^	3
	EXOCAD	3

### 2.1 Study design and patient selection

Participants who had implant surgery designed by the GuideMia implant system or 3Shape implant studio by the same clinician (S.K) between March 2020 and March 2023 were consecutively selected and enrolled. Data from 75 participants who met the inclusion and exclusion criteria were all collected in this retrospective study. A total of 75 implants were inserted, and 40 in the GuideMia group and 35 in the 3Shape group. Then a *post-hoc* analysis of the sample size was performed, revealing a statistical power of 0.66. The retrospectively registration number is ChiCTR2400080259 and the date of registration is January 24, 2024.

The inclusion criteria were as follows: (1) Single missing tooth in the molar or premolar area (maxilla or mandible); (2) Postoperative CBCT scan had already been performed; (3) The number of oral scans was <1000; (4) Sufficient bone in the edentulous area at the time of surgery and without bone grafting; (5) Well-aligned dentition; and (6) The implanted implant were bone-level and round apical and showed good primary stability immediately after placement (IOS > 60).

The exclusion criteria were as follows: (1) Untreated or active periodontal disease; (2) Smoking >10 cigarettes/day; (3) Presence of chronic systemic disease; and (4) With metal restorations.

### 2.2 Data acquisition

**Figure 1:**
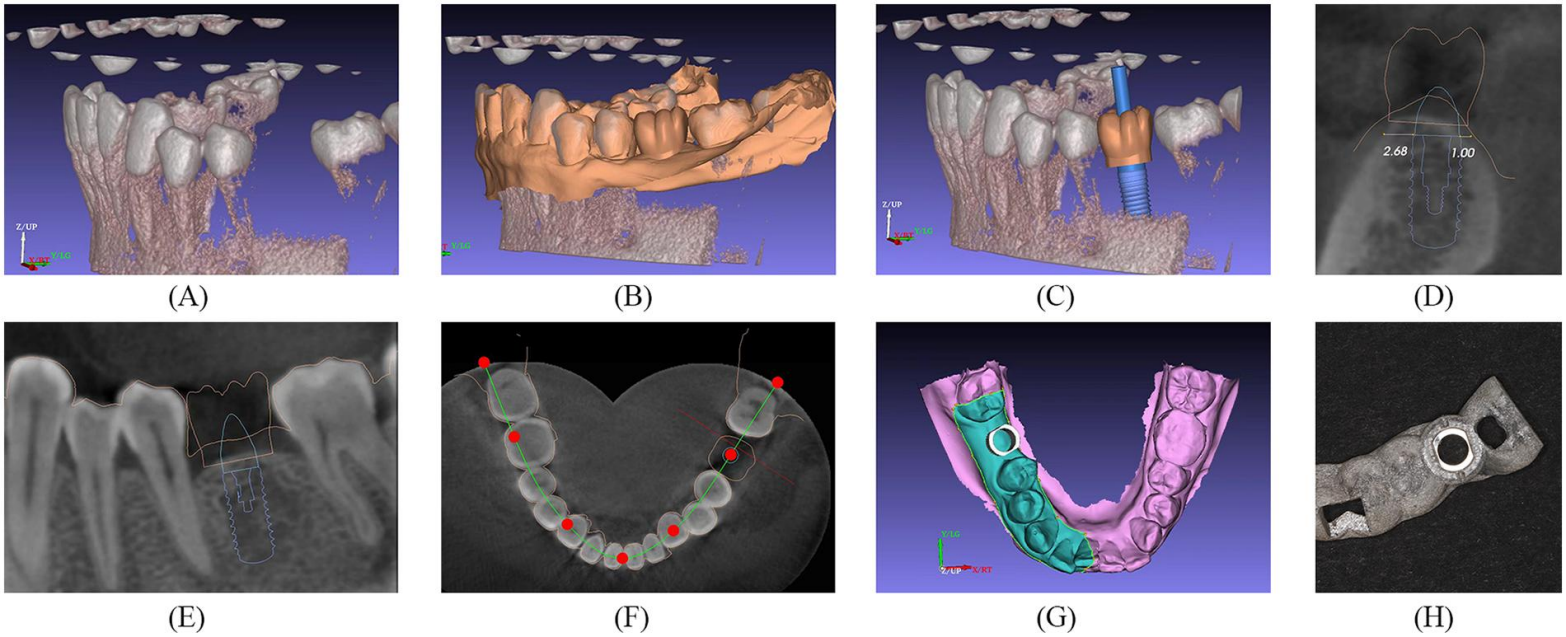
Design of the surgery-guided template in the GuideMia implant system. **(A)** Preoperative CBCT scans were imported into the program package. **(B)** IOS data were matched to CBCT images, and a virtual wax-up was created. **(C)** The implant type and size were selected from the implant library. **(D)** Planned implant placement (coronal view). **(E)** Planned implant placement (sagittal view). **(F)** Planned implant placement (cross view). **(G)** Drawing guide range. **(H)** 3D printing guide.

**Figure 2:**
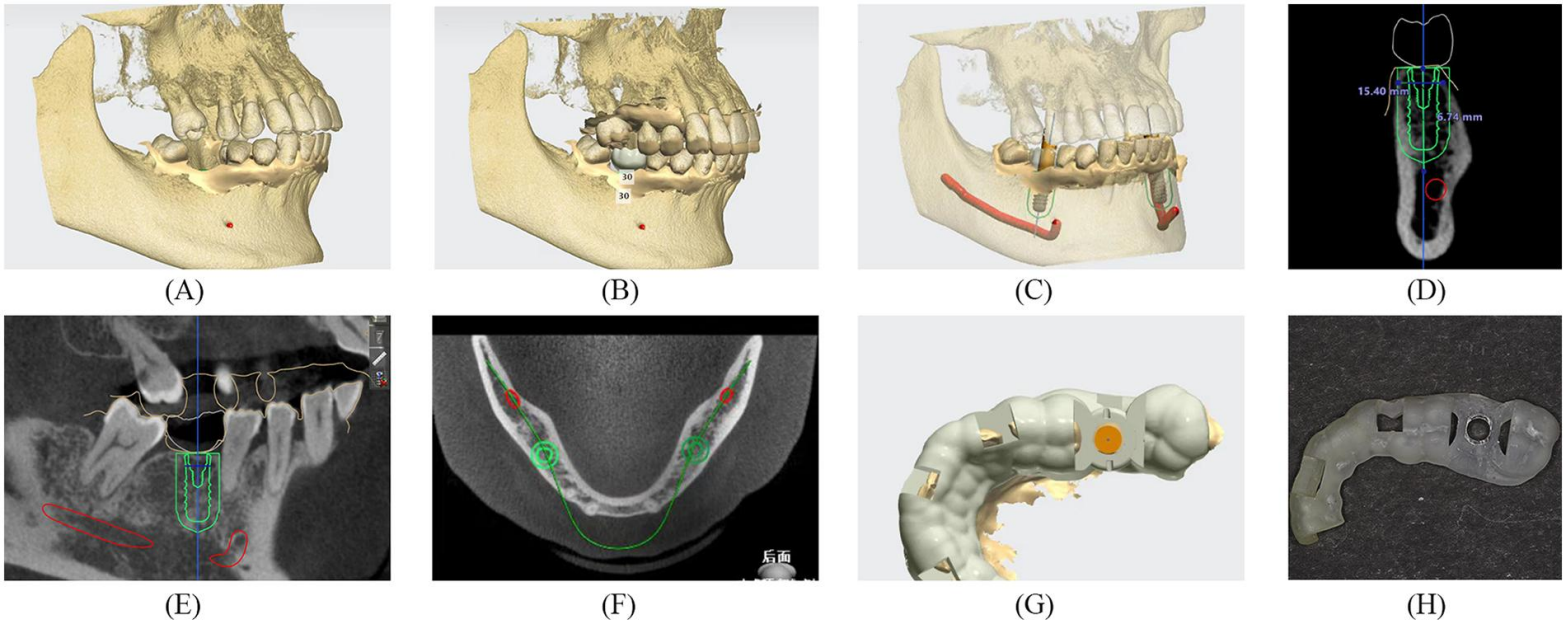
Design of the surgery-guided template in the 3Shape implant studio. **(A)** Preoperative CBCT scans were imported into the program package. **(B)** IOS data were matched to CBCT images, and a virtual wax-up was created. **(C)** The implant type and size were selected from the implant library. **(D)** Planned implant placement (coronal view). **(E)** Planned implant placement (sagittal view). **(F)** Planned implant placement (cross view). **(G)** Drawing guide range. **(H)** 3D printing guide.

After the initial examination, all patients whose teeth were separated by a cotton roll underwent a CBCT scan (KaVo Dental, Germany) under exposure parameters (120 kV, 5 mA, 26.9 s, FOV: 160 mm × 130 mm, voxel size: 250 µm) to confirm the presence of adequate bone volume for an implant placement. Surface scans of oral tissues were performed using a Trios intraoral scanner (3 Shape TRIOS, Denmark). Unprocessed raw CBCT scans in the Digital Imaging and Communications in Medicine (DICOM) format and IOS data in the Standard Tesselation Language (STL) format were directly imported into the implant planning program. The CBCT data were segmented to remove artifacts and excess undesirable tissue, resulting in a virtual 3D model that exclusively showed the teeth and bone. Several common landmarks or reference points that could be easily identified in both the STL files and CBCT images and located in the area of interest were defined. The automatic alignment algorithm within the software was used to calculate the best-fit alignment based on the defined landmarks. A virtual wax-up was used to simulate the final restoration of the digital model, considering the direction of the occlusal force, shape of the axial surface, and adjacency relationship. After creating a “digital patient” that included the tooth, underlying bone, and digital prosthetic, the implant type and size were selected from the implant library, following the prosthetic-driven concept. There was a safe distance of 2.0 mm from the inferior alveolar nerve. The same clinician performed all implant-planning steps. Once the planning phase was completed, the designed surgical guide was exported to the STL format for fabrication. A detailed report was generated, including the drilling protocol and corresponding implant. The surgical guide with inspection window was printed using a 3D printer (Evo DentDLP S110), and the implant system-specific metal sleeve (Ø 5 mm or Ø 3.7 mm T-sleeves) was subsequently incorporated (Figures 1, 2). All guides were extended to cover 4–6 teeth.

### 2.3 Surgical protocol

**Figure 3:**
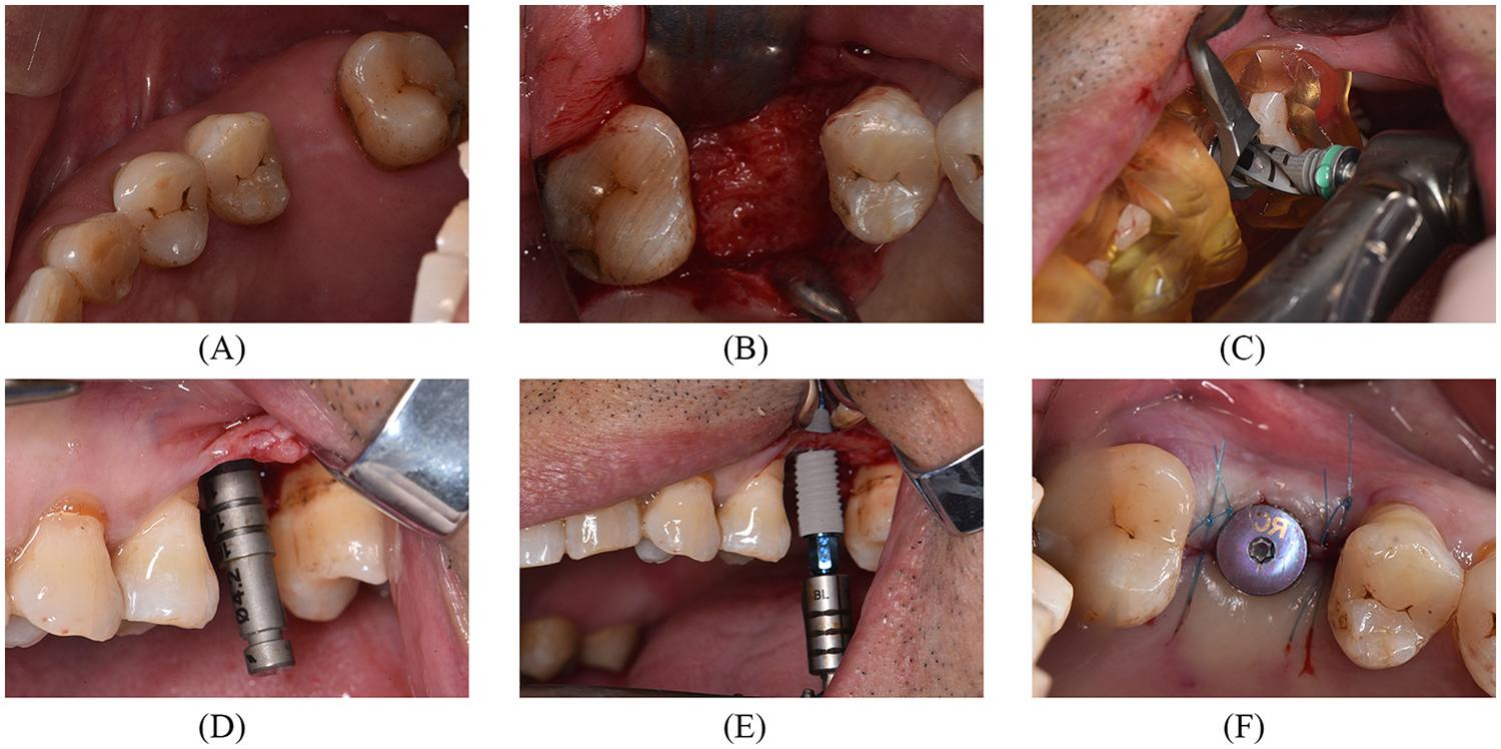
Surgical process with partial guided protocol**. (A)** Preoperative occlusal view. **(B)** Occlusal view after the open flap. **(C)** Implant drill sequence with the surgical guide. **(D)** The direction of the prepared hole. **(E)** The implant (Straumann BL, Switzerland) was inserted after continuous drilling. **(F)** Install healing abutment.

All surgeries were performed using a partially guided protocol by the clinician who guided the preoperative implant planning, meaning that the surgical guide was used only for drilling procedures (13). Before the surgical treatment, the tooth-supported surgical drill guide was seated properly. Open-flap implant placement was performed, and the recommended guided surgical drill sequence derived from the manufacturer was used to perform the osteotomy. Transmucosal healing occurred in all implants that were placed as one-stage implants (Figure 3). Patients were instructed to consume a soft diet for 1 week and avoid brushing the surgical sites. They were also prescribed ibuprofen 600 mg every 6 h for the first 48 h to manage any potential pain and were instructed to rinse their mouths twice daily with a chlorhexidine mouthwash for 2 weeks post-surgery. Implant diameters ranged from 3.3 to 4.8 mm, and lengths of 8, 10, and 12 mm were used (Institut Straumann AG, Basel, Switzerland, and Axiom REG France).

### 2.4 Accuracy evaluation *in vivo*

**Figure 4:**
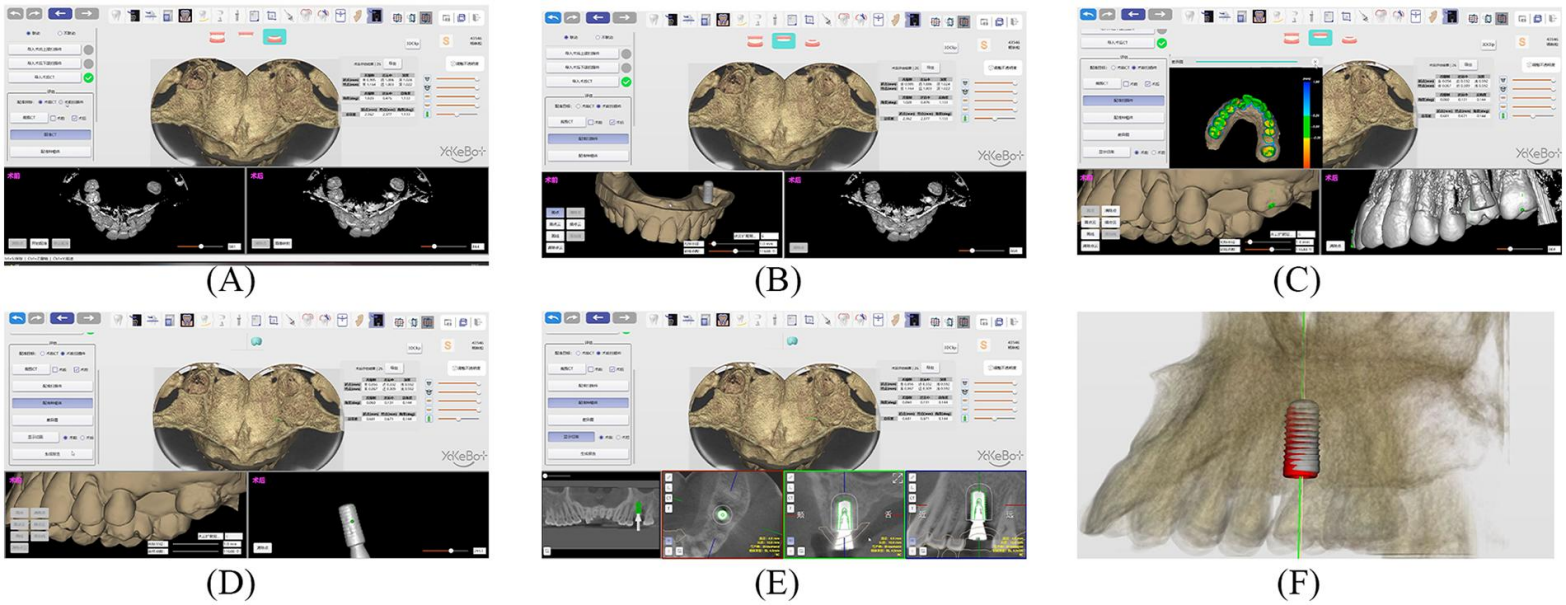
**(A,B)** Postoperative and preoperative CBCTs with virtual planning were imported into dentalNavi. **(C,D)** Registration of scans and implants. **(E,F)** The 3D view of the preoperative plan compared with postoperative CT.

**Figure 5:**
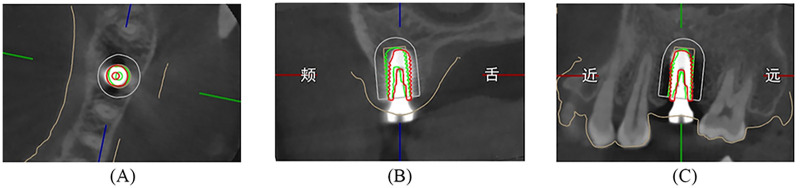
**(A–C)** Postoperative evaluation: comparing the accuracy of preoperative (green) with postoperative actual implant position (red).

**Figure 6:**
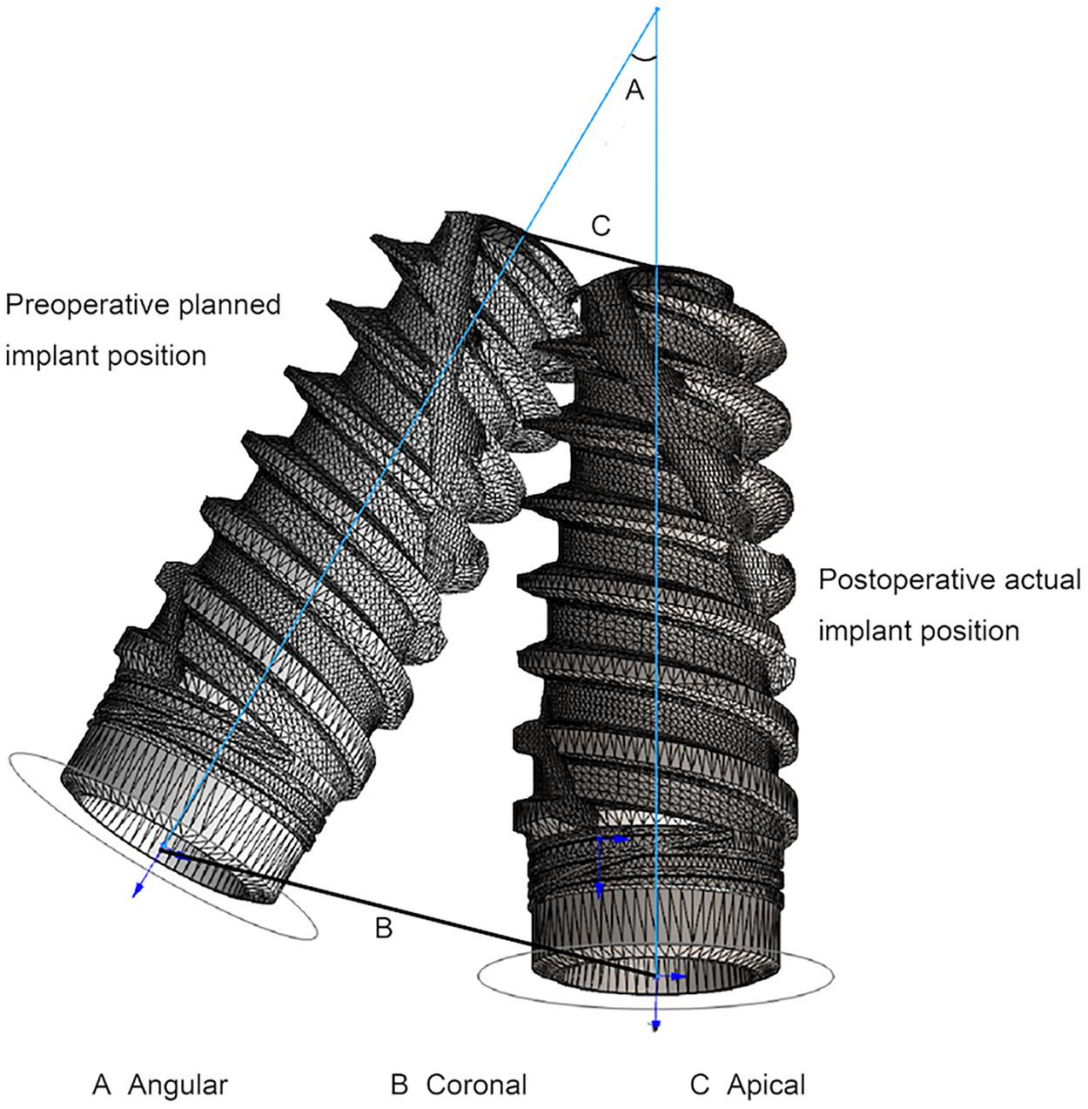
Schematic diagram of the deviation between planned and actual implant positions.

A new CBCT scan (120 kV, 5 mA, 26.9 s, FOV: 160 mm × 130 mm, voxel size: 250 µm, slice thickness:0.25 mm) was made immediately after surgery. The DICOM files originating from the post- and preoperative CBCT with virtual planning were imported into an extra evaluation software program (DentalNavi; YakeRobot Technology Ltd) (23). The alignment process was initiated by selecting the “Align CT” function. This involved identifying and selecting three evenly distributed common landmarks or reference points that were easily identifiable in both CBCT images to complete the registration. Thereafter, the “Align Implant” function was used by selecting the implant image in the postoperative CBCT scan. The process was concluded by clicking “Generate Report” and the software generated a projection of the preoperative position vs. the postoperative position, resulting in an electronic report of the 3D deviation measurement analysis for each patient (Figures 4, 5). This alignment process employed the image best-fit algorithm [ICP (Iterative Closest Point) surface registration]. For accuracy evaluation, the following three outcome parameters were recorded: coronal and apical 3D distance deviation in millimeters and angular deviation of the implant in degrees (Figure 6). Coronal deviation was defined as the distance from the center of the planned implant position entry point to the center of the new implant position entry point. Apical deviation was defined as the distance from the center of the planned implant position to the center of the new implant position. Angular deviation was defined as the angle between the long axes of the implant. All cases were processed thrice by the same investigator, and the average value was used. Additionally, the intra-class correlation coefficient (ICC) values for these measurements all exceeded 0.9, indicating excellent reliability.

The predictor risk factors that may separately affect the accuracy of the guided implant surgery were defined and categorized as follows: implant length (8, 10, 12 mm), implant diameter (3.3, 4, 4.1, 4.6, 4.8 mm), the location of the implant site (premolar or molar), the jaw position of the implant site (maxilla or mandible), implant system (Straumann BL or Axiom REG), and missing tooth at free end (Yes, No).

### 2.5 Statistical analysis

All statistical analyses were performed using SPSS for Windows Release 27 software (SPSS Inc., Chicago, IL, USA). The alpha level was set at 0.05. Accuracy data were analyzed at the implant level, and the mean (±) standard deviation was reported. A two-tailed independent *t*-test and a one-way analysis of variance were performed for the univariate analysis of risk factors with two variables and three or more variables, respectively. The risk factors of the jaw, location, system, diameter, and length of the implants were often related; thus, multiple linear regression analysis was performed to determine the impact of different risk factors on deviations. The influence of the categorical variables was incorporated into the regression model by setting dummy variables.

## 3 Results

### 3.1 Patient information

In total, 59 implants were placed in the mandible and 16 in the maxilla. Of these, 49 were from the Straumann BL system, and 26 were from the Axiom REG system. The implant lengths were 8, 10, and 12 mm in 16, 49, and 10 patients, respectively. The implant diameters were 3.3, 4, 4.1, 4.6, and 4.8 mm for 4, 7, 15, 16, and 33 patients, respectively. Additionally, 20 implants were placed in the premolars, 55 in the molars, and 22 implants were placed in the free end position. Table 2 presents the characteristics of the implants and the position of implant placement. No statistically significant differences were found in these variables between the two groups (*P* > 0.05).

**Table 2:** Characteristics and position of placed implants between the guideMia and 3Shape systems.

Characteristics	GuideMia	3Shape	Total	*P*
Implant's diameter	—	—	—	0.853^b^
Narrow (3.3 mm)	1	3	4	—
Regular (4 mm)	7	3	7	—
Regular (4.1 mm)	5	7	15	—
Regular (4.6 mm)	15	8	16	—
Regular (4.8 mm)	12	21	33	
System	—	—	—	0.163^a^
Straumann	20	29	49	
Axiom	15	11	26	
Implant's length	—	—	—	0.921^b^
8 mm	7	9	16	—
10 mm	28	21	49	—
12 mm	5	5	10	—
Free end missing	—	—	—	0.378^a^
Yes	10	12	22	—
No	30	23	53	—
Jaw	—	—	—	0.407^a^
Maxilla	10	6	16	—
Mandible	30	29	59	—
Position	—	—	—	0.222^a^
Premolar	13	7	20	—
Molar	27	28	55	—

a Differences between groups were analyzed using Pearson's chi-squared test.

b Differences between groups were analyzed using the Fisher exact test.

### 3.2 Implant deviation comparison

In the GuideMia group, the average coronal, apical, and angular deviations were 1.16 mm, 1.79 mm, and 3.60° respectively for implants with a length of 8 mm. For implants with a length of 10 mm, the deviations were 1.10 mm, 1.20 mm, and 4.45°. And for implants with a length of 12 mm, the deviations were 0.95 mm, 1.38 mm, and 5.25°. In the 3Shape group, the corresponding deviations were 0.88 mm, 1.27 mm, and 3.96° respectively for implants with a length of 8 mm. For implants with a length of 10 mm, the deviations were 1.06 mm, 1.08 mm, and 3.67°. And for implants with a length of 12 mm, the deviations were 0.59 mm, 0.80 mm, and 3.13°. No statistically significant differences were found in these variables between the two implant planning software programs, regardless of implant length. (*P* > 0.05) [Table 3(1,2,3)].

**Table 3:** Deviation Of actual implant position and the planned implant position between the GuideMia and 3Shape systems.

Group	*N*	Coronal deviation (mm)	Apical deviation (mm)	Angular deviation (°)
(1) 8 mm length implant				
GuideMia	7	1.16 ± 0.34	1.79 ± 0.66	3.60 ± 1.92
3Shape	9	0.88 ± 0.46	1.27 ± 0.51	3.96 ± 2.20
*t*-value		1.30	1.76	0.34
*p*-value		0.21	0.09	0.73
(2) 10 mm length implant				
GuideMia	28	1.10 ± 0.42	1.20 ± 0.59	4.45 ± 2.49
3Shape	21	1.06 ± 0.72	1.08 ± 0.51	3.67 ± 2.67
*t*-value		0.22	0.73	1.05
*p*-value		0.82	0.46	0.29
(3) 12 mm length implant				
GuideMia	5	0.95 ± 0.72	1.38 ± 0.75	5.23 ± 2.77
3Shape	5	0.59 ± 0.13	0.80 ± 0.21	3.13 ± 2.16
*t*-value		1.08	1.64	1.33
*p*-value		0.33	0.13	0.21

### 3.3 Risk factor analysis

Some significant differences were found in the multiple linear regression analysis of individual risk factors for guided implant placement accuracy. First, all models satisfied the linearity, independence, homoscedasticity, and normality of the residuals. The R Square value was 0.163 for coronal deviations, 0.164 for angular deviations, and 0.211 for apical deviations, indicating a better fit of the models to the data. The results showed that independent variables, such as the implanted jaw, implant system, implant position, diameter, and length of the implant, had no significant influence (*P* > 0.05). However, a significant difference was found at the implant entry point (*P* = 0.003), apex (*P* = 0.005), and angle (*P* = 0.002) between the free end and non-free end implant placements. All risk factors and subsequent mean results are summarized in Table 4.

**Table 4:** Results of the multivariate analysis per risk factor.

Risk factor	*N*	Coronal deviation (mm)			Apical deviation (mm)			Angular deviation (°)		
		Mean	SD	*P*	Mean	SD	*P*	Mean	SD	*P*
Implant's diameter				0.54			0.34			0.06
Narrow (3.3 mm)	4	1.16	0.78		1.15	0.60		4.11	3.34	
Regular (4 mm)	7	1.21	0.67		1.05	0.57		5.26	1.64	
Regular (4.1 mm)	15	0.83	0.50		1.09	0.54		2.71	0.74	
Regular (4.6 mm)	16	1.05	0.40		1.18	0.53		3.56	2.09	
Regular (4.8 mm)	33	1.05	0.56		1.20	0.62		4.64	2.88	
System				0.30			0.40			0.95
Straumann	49	0.98	0.57		1.17	0.59		3.95	2.74	
Axiom	26	1.12	0.46		1.29	0.59		3.91	2.15	
Implant's length				0.23			0.09			0.90
8 mm	16	1.00	0.42		1.50	0.62		3.68	2.12	
10 mm	49	1.09	0.56		1.15	0.56		4.01	2.66	
12 mm	10	0.77	0.52		1.09	0.60		4.98	2.76	
Free end missing				0.003			0.005			0.002
Yes	22	1.31^a^	0.64		1.51^a^	0.72		5.37^a^	3.08	
No	53	0.91^a^	0.44		1.09^a^	0.48		3.51^a^	1.90	
Jaw				0.70			0.30			0.84
Maxilla	16	0.98	0.60		1.08	0.63		4.05	3.39	
Mandible	59	1.04	0.52		1.25	0.57		3.91	2.29	
Position				0.62			0.44			0.57
Premolar	20	1.08	0.63		1.30	0.62		3.79	2.13	
Molar	55	1.01	0.50		1.18	0.58		4.15	2.55	

a Significantly different in the multivariate analysis.

## 4 Discussion

Implant planning software can effectively assist clinicians in identifying the optimal location for implants following the “prosthetic-driven” principle and accurately deciding for surgery through static surgical guides or dynamic navigation (24, 25). Our results showed no significant differences in the clinical accuracy of the planned and placed positions for implants of the same length between the two software packages. Thus, the null hypothesis is accepted. According to recent systematic reviews and meta-analysis of clinical studies, the mean deviation in the entry point position of implants was approximately 1.1–1.4 mm, apical deviation was approximately 1.2–1.6 mm, and angle deviation was approximately 3.0°–4.3° (26, 27). Our results are consistent with those of previous studies. The deviation in the apical direction was 1.0–1.5 mm for the two software; thus, a vertical safety distance of 2 mm should be sufficient.

However, the 3Shape Implant Studio and GuideMia implant system have advantages and limitations. GuideMia primarily focuses on implant surgery planning and execution and provides comprehensive tools for virtual implant placement and surgical guide design. 3Shape is compatible with intraoral scanners and offers a broad range of dental CAD/CAM capabilities, including digital impression scanning, dental laboratory design, and guide production solutions (15, 28). The choice between these two depends on specific requirements and user preferences. Clinicians who primarily focus on implant dentistry may find GuideMia more suitable for their specialized needs. Those seeking a more comprehensive dental CAD/CAM solution may prefer the 3Shape. Second, the GuideMia has a steep learning curve. This is because of the complex user interface and workflow, which require users to navigate through multiple menus, tools, and options to perform specific tasks, particularly during the guide design process. Several critical decisions regarding virtual implant placement must be made manually. This complexity poses a challenge for beginners who may need to dedicate time and effort to become proficient in efficiently using the software. In contrast, 3Shape offers a user-friendly interface that is intuitive and easy to operate. It offers a simplified workflow that smoothly guides the users in each step by providing clear instructions and prompts. Third, the cost of GuideMia and 3Shape software varies based on the specific package, modules, and licensing options chosen. GuideMia is affordable, making it a popular choice for smaller dental practices. 3Shape, a comprehensive CAD/CAM solution, generally comes at a higher cost. Additionally, both are compatible with various implant systems, facilitating collaboration between dental laboratories, clinicians, and implant manufacturers. In summary, clinicians should carefully evaluate specific needs and consider the relevant factors when deciding between them (29, 30). Notably, these differences may evolve over time as software companies update their products and add new features. The primary strength of the research lies in the objective comparison of two implant design systems via independent third-party software, which effectively mitigated measurement bias and ensured a fair evaluation of the deviations in each system. The limitations of this study are that the baseline data of the two groups were not completely standardized and the inherent bias in retrospective studies. This may have introduced potential biases when accurately comparing the performance of different software packages. Future studies should focus on establishing strict inclusion criteria by considering factors such as varying bone densities, different implant sites, and levels of operator experience to obtain reliable and meaningful results. Furthermore, although single-operator control reduces variability in surgical techniques and ensures consistency in procedural workflows, it may also introduce systematic bias because the surgical outcomes can be influenced by the personal experience, skill level, and subjective judgment of the operator. Future studies could involve multiple operators to increase the generalizability of the results and to further validate our findings across various clinical settings.

Variability in the accuracy of guided implant surgery has been reported to be primarily because of errors originating from intrinsic and extrinsic sources (31–33).Intrinsic errors refer to inaccuracies in the design or fabrication of the guide, errors in software algorithms, limitations in imaging technology, or registration matching errors of CBCT and IOS data. Extrinsic errors are related to the operator's technique, patient anatomy, and guide during surgery. In this study, we included patients with well-aligned dentition, without any metal restorations, and with high accuracy in intraoral scans. A skilled clinician performed all steps to minimize preoperative design errors. An accuracy advantage was observed for non-free end single-implant placement cases. This finding suggests that free end sites may result in increased complexity in realizing the ideal position of the implant, owing to the absence of adjacent teeth for support and guidance. In contrast, missing non-free ends can offer additional stability and reference points for implant positioning. According to López et al. (34), there may be slight movements of the surgical guide during the drilling process, and using a guide supported on one side may result in larger implant deviations owing to the tilting and bending of the guide. El Kholy et al. (35) reported in an *in vitro* study that implants placed in a distal free end position exhibited significantly greater entry points and apical deviations than did implants placed using a bilateral tooth-supported guide. This reveals that when designing implant placement at the free end, stability can be enhanced by improving the accuracy of the surgical guide to ensure proper fit with the dental arch or by covering more teeth to achieve maximized support from neighboring teeth (36). Additionally, longer implants can be more challenging to place accurately due to variations in bone density and quality along the implant site, making it difficult to achieve optimal placement. And longer implants may require additional surgical skill and experience to properly place, as the increased length can make it more challenging to control the position and angulation of the implant during placement. However, the risk factor analysis in this study did not find any impact of changes in implant length on accuracy. This may be due to the small sample size, indicating a need for further research with a larger sample to validate these findings. Furthermor, the limitations of pre- and post-operative CBCT superimposition must be considered. First, metal artifacts from implants and abutments can obscure the bone-implant interface, compromising the precision of axis identification and potentially introducing measurement bias. Second, alveolar remodeling and extractions reduce the stable surface area required for accurate registration. Finally, the inherent registration errors (0.1–0.5 mm) inherent in surface-based algorithms, which may mask minor deviations between systems.

In this study, round apical design implants were used, which may have a higher risk of being pushed away by the crestal bone or cortical wall, potentially leading to inaccuracies in the implant position. Conversely, implants with a more aggressive apical thread design may improve engagement with denser cortical bone, enhancing stability and accuracy during placement (37). Furthermore, many researchers indicated that the accuracy of fully guided templates is higher than that of partially guided templates (13, 38, 39). Therefore, additional experiments are necessary to compare and evaluate the deviation in accuracy between the different guidance methods.

Although the application of CAD software has shown positive results, certain deviations remain. Therefore, improving the performance of various types of equipment and software is crucial. As research on full digital workflow deepens, more precise data acquisition equipment will become available, and clinicians will continuously improve their skills in controlling errors at each step of the digital workflow.

## 5 Conclusions

No significant differences were found in the accuracy of implant position between the GuideMia implant system and the 3Shape Implant Studio program. Clinicians should carefully evaluate their specific needs and consider the relevant factors in clinical application. Additionally, implants located at the free end of the dental arch, which lacked bilateral neighboring teeth to support the drill guide, exhibited larger deviations at the implant apices and entry points. Further improvement of the full digital workflow, leading to higher placement accuracy, is recommended.

## Data Availability

The raw data supporting the conclusions of this article will be made available by the authors, without undue reservation.
